# Ray Statement of the Acoustic Tomography Problem

**DOI:** 10.1134/S1064562422040147

**Published:** 2022-09-27

**Authors:** V. G. Romanov

**Affiliations:** Sobolev Institute of Mathematics, Siberian Branch, Russian Academy of Sciences, 630090 Novosibirsk, Russia

**Keywords:** acoustic equation, acoustic tomography, ray expansion, inverse kinematic problem, integral geometry

## Abstract

The ray statement of the inverse problem of determining three unknown variable coefficients in the linear acoustic equation is studied. These coefficients are assumed to differ from given constants only inside some bounded domain. There are point pulse sources and acoustic receivers on the boundary of this domain. Acoustic signals are measured by a receiver near the moment of time at which the signal from a source arrives at the receiver. It is shown that this information makes it possible to uniquely determine all the three desired coefficients. Algorithmically, the original inverse problem splits into three subproblems solved successively. One of them is a well-known inverse kinematic problem (of determining the speed of sound), while the other two lead to the same integral geometry problem for a family of geodesic lines determined by the speed of sound.

Acoustic tomography problems were posed rather long ago (see, for example, [1–4]). Initially, they were motivated by the use of sound waves for early diagnosis of breast cancer. Recently, due to the spread of COVID-19 infection, the use of acoustic tomography methods has been proposed for diagnosing lung diseases as well. Computational modeling of acoustic tomography problems was carried out in [5–9] (see also numerous references therein). Below, we consider one of the possible variants for posing an acoustic tomography problem. In this problem, point pulse sources and receivers of acoustic signals are located outside the domain in which the variable coefficients in the acoustic equation are sought. Acoustic signals are measured in a small neighborhood of the moment of time at which the signal from a source arrives at the corresponding receiver. This problem belongs to the class of ray inverse problems introduced by the author in [10]. A characteristic feature of problems of this type is that the original problem of determining several coefficients is split into several successively solved problems of determining one of the required coefficients. In our case, these sought coefficients are the speed of sound *c*(*x*), the sound absorption coefficient $\sigma (x)$, and the medium density $\rho (x)$. Determining *c*(*x*) reduces to solving a well-known inverse kinematic problem, whereas determining $\sigma (x)$ and $\rho (x)$ leads to solving some integral geometry problem for a family of geodesic lines for the conformal Riemannian metric specified by the coefficient *c*(*x*). In the case when $c(x) \equiv 1$, this problem is a common X-ray tomography problem.

We consider the Cauchy problem

1 $$\begin{gathered} {{c}^{{ - 2}}}(x){{p}_{{tt}}} + \sigma (x){{p}_{t}} - \Delta p - \nabla \ln \rho (x) \cdot \nabla p \\ \, = \delta (x - y)\delta (t), \quad (x,t) \in {{\mathbb{R}}^{4}}; \quad p{{{\text{|}}}_{{t < 0}}} = 0, \\ \end{gathered} $$

where $p = p(x,t)$ is the sound pressure, $c(x) > 0$ is the speed of sound, $\sigma (x) \geqslant 0$ is the sound absorption, and $\rho (x)$ > 0 is the medium density. Equation (1) describes the propagation of acoustic waves in an inhomogeneous absorbing medium. Acoustic tomography is based on this equation. The main acoustic tomography problem is to solve an inverse problem for Eq. (1), namely, to construct the coefficients $c(x)$, $\sigma (x)$, and $\rho (x)$ inside a bounded domain based on information given outside this domain, namely, the solutions of Eq. (1) on some interval [0, *T*] for a set of point sources *y*. For definiteness, the following model is considered below.

Let ${{B}_{R}}$ be the ball of radius *R* centered at the origin, and let ${{S}_{R}}$ be its boundary. We assume that the parameters $c(x),\;\sigma (x),\;\rho (x)$ are continuously differentiable functions everywhere in ${{\mathbb{R}}^{3}}$ (see below for additional conditions (9)); they are unknown inside a ball ${{B}_{{{{r}_{0}}}}}$, $R > {{r}_{0}} > 0$, and are given constants outside it:

2 $$\begin{gathered} c(x) = 1, \quad \sigma (x) = 0, \\ \rho (x) = {{\rho }_{0}} > 0,\quad x \in {{\mathbb{R}}^{3}}{{\backslash }}{{B}_{{{{r}_{0}}}}}. \\ \end{gathered} $$

Let ${{S}_{R}}(y,{{r}_{0}})$ denote the projection of the ball ${{B}_{{{{r}_{0}}}}}$ onto the sphere ${{S}_{R}}$ around the source located at $y \in {{S}_{R}}$, that is, ${{S}_{R}}(y,{{r}_{0}})$ = $\{ x \in {{S}_{R}}\,{\text{|}}\,{\text{|}}x - y{\text{|}} \geqslant 2\sqrt {{{R}^{2}} - r_{0}^{2}} \}$. We denote the solution of problem (1) by $p(x,t,y)$, thus underlying its dependence on the parameter *y*. We fix $y \in {{S}_{R}}$ and $x \in {{S}_{R}}(y,{{r}_{0}})$. Let *T*_0_(*x*, *y*) := sup{τ > 0 | $p(x,t,y) \equiv 0$, $t \in (0,\tau )\}$.

We consider the following statement of the acoustic tomography (AT) problem.

**AT problem.** Assume that $p(x,t,y)$ is a solution of problem (1) and the function

3 $$\begin{gathered} F(x,t,y) = p(x,t,y)\quad {\text{for}}\,\,{\text{all}}\quad y \in {{S}_{R}}, \\ x \in {{S}_{R}}(y,{{r}_{0}}),\quad t \in (0,{{T}_{0}}(x,y) + \varepsilon ), \\ \end{gathered} $$

where $\varepsilon > 0$ is fixed and arbitrarily small, is known. We need to find $c(x)$, $\sigma (x)$, and $\rho (x)$ inside ${{B}_{{{{r}_{0}}}}}$ given $F(x,t,y)$.

Introducing the new function *u*(*x*, *t*, *y*) = $p(x,t,y){{\rho }^{{1/2}}}(x)$, we reduce problem (1) for $y \in {{S}_{R}}$ to the form

4 $$\begin{gathered} Lu \equiv {{c}^{{ - 2}}}(x){{u}_{{tt}}} + \sigma (x){{u}_{t}} - \Delta u + q(x)u \\ \, = \rho _{0}^{{1/2}}\delta (x - y)\delta (t),\quad (x,t) \in {{\mathbb{R}}^{4}}; \quad u{{{\text{|}}}_{{t < 0}}} = 0, \\ \end{gathered} $$

where the coefficient *q*(*x*) is given by the formula

5 $$q(x) = {{\rho }^{{ - 1/2}}}(x)\Delta {{\rho }^{{1/2}}}(x).$$

If the coefficient *q*(*x*) is given, then, as follows from (5), the function $m(x) = {{\rho }^{{1/2}}}(x)$ can be obtained inside ${{B}_{{{{r}_{0}}}}}$ as a solution of the Dirichlet problem

6 $$\Delta m(x) - q(x)m(x) = 0,\quad x \in {{B}_{{{{r}_{0}}}}};\quad m{{{\text{|}}}_{{r = {{r}_{0}}}}} = \rho _{0}^{{1/2}},$$

where $r = {\text{|}}x{\text{|}}$. In addition, condition (2) on the function $\rho (x)$ and its continuous differentiability everywhere in ${{\mathbb{R}}^{3}}$ imply that the solution of problem (6) must satisfy the requirement $\frac{{\partial m}}{{\partial r}} = 0$ at $r = {{r}_{0}}$. This additional condition guarantees the uniqueness of determining *m*(*x*) and, consequently, $\rho (x)$ from the given *q*(*x*). Therefore, instead of the problem of determining $c(x)$, $\sigma (x)$, and $\rho (x)$ in ${{B}_{{{{r}_{0}}}}}$, it is convenient to consider the inverse problem of finding the coefficients $c(x)$, $\sigma (x)$, and *q*(*x*) for (4) in the same domain based on information similar to (3), namely,

7 $$\begin{gathered} \Phi (x,t,y) = u(x,t,y)\quad {\text{for}}\,\,{\text{all}}\quad y \in {{S}_{R}}, \\ x \in {{S}_{R}}(y,{{r}_{0}}),\quad t \in (0,{{T}_{0}}(x,y) + \varepsilon ). \\ \end{gathered} $$

Here, $\Phi (x,t,y) = F(x,t,y)\rho _{0}^{{1/2}}$ and ε is fixed and arbitrarily small.

Similar problems were considered in [10] for $x \in {{\mathbb{R}}^{2}}$ in the following cases:

(1) $c(x) \equiv 1$, while $\sigma (x)$ and *q*(*x*) are unknown [10, Subsection 5.2],

(2) $\sigma (x) \equiv 0$, while $c(x)$ and *q*(*x*) are unknown [10, Subsection 5.3].

In addition, in [10, Subsection 5.4], a two-dimensional inverse problem in electrodynamics is considered. When the properties of the medium are independent of ${{x}_{3}}$, the equation for the electric field component ${{E}_{3}}$ coincides with Eq. (1), in which the role of $\rho (x)$ is played by the magnetic permeability of the medium and the coefficient $\sigma (x)$ specifies the electric conductivity of the medium. In this inverse problem, all three unknown coefficients are determined based on dynamical information on solutions of three Cauchy problems in which the point source $\delta (x - y)$ is replaced by the plane wave source $\delta (x \cdot {{\nu }^{{(k)}}})$, where ${{\nu }^{{(k)}}}$ is a unit vector and *k* = 1, 2, 3.

We consider a Riemannian metric such that the length element $d\tau$ is defined as $d\tau = {\text{|}}dx{\text{|/}}c(x)$, where ${\text{|}}dx{\text{|}} = {{\left( {\sum\limits_{k = 1}^3 {{{{(d{{x}_{k}})}}^{2}}} } \right)}^{{1/2}}}$. Throughout the rest of this paper, we assume that the following assumption holds.

**Assumption.** The Riemannian metric *d*τ = ${\text{|}}dx{\text{|/}}c(x)$ is simple in ${{\mathbb{R}}^{3}}$, that is, any two points *x* and *y* in ${{\mathbb{R}}^{3}}$ can be connected by a unique geodesic line $\Gamma (x,y)$.

Note that a sufficient condition for the simplicity of a conformal Riemannian metric in ${{\mathbb{R}}^{3}}$ is the condition (see [11])

$$\sum\limits_{i,j = 1}^3 \frac{{{{\partial }^{2}}\left( {\ln c(x)} \right)}}{{\partial {{x}_{i}}\partial {{x}_{j}}}}{{\xi }_{i}}{{\xi }_{j}} \leqslant 0,\quad \forall \xi ,\quad x \in {{\mathbb{R}}^{3}},$$

where $$\xi = ({{\xi }_{1}},{{\xi }_{2}},{{\xi }_{3}})$$.

**Remark 1.** In fact, within the ray statement of the inverse problem, the above assumption can be weakened, namely, the Riemannian metric can be assumed to be simple only inside a fixed ball ${{B}_{{R'}}}$ for $R' > R$. We have limited ourselves to a stronger assumption to avoid extra remarks in what follows.

Assume that there are finite numbers ${{c}_{0}}$ and ${{c}_{{00}}}$ such that

8 $$0 < {{c}_{0}} \leqslant c(x), \quad x \in {{\mathbb{R}}^{3}};\quad {{\left\| c \right\|}_{{{{C}^{2}}({{\mathbb{R}}^{3}})}}} \leqslant {{c}_{{00}}},$$

and that the coefficients in (4) are characterized by the following smoothness:

9 $$c \in {{C}^{6}}({{\mathbb{R}}^{3}}), \quad \sigma \in {{C}^{4}}({{\mathbb{R}}^{3}}), \quad q \in {{C}^{2}}({{\mathbb{R}}^{3}}).$$

Let $\tau (x,y)$ denote the Riemannian distance between points *x* and *y*. Physically,$\tau (x,y)$ is the travel time of the acoustic signal between *x* and *y*. It is well known that $\tau (x,y)$ is a solution of the following Cauchy problem for the eikonal equation:

10 $${\text{|}}{{\nabla }_{x}}\tau (x,y){{{\text{|}}}^{2}} = \frac{1}{{{{c}^{2}}(x)}};\quad \tau (x,y) \sim \frac{{{\text{|}}x - y{\text{|}}}}{{c(y)}},\quad x \to y.$$

To find $\tau (x,y)$ and derive equations of geodesic lines, we need to introduce the vector $\eta = {{\nabla }_{x}}\tau (x,y)$ and to solve the following Cauchy problem for the Euler system of ordinary differential equations:

11 $$\frac{{d\xi }}{{ds}} = \eta (\xi ){{c}^{2}}(\xi ),\quad \frac{{d\eta (\xi )}}{{ds}} = - \nabla \ln c(\xi ),\quad \frac{{d\tau }}{{ds}} = 1,$$

12 $$\xi {{{\text{|}}}_{{s = 0}}} = y,\quad \eta {{{\text{|}}}_{{s = 0}}} = \frac{\nu }{{c(y)}},\quad \tau {{{\text{|}}}_{{s = 0}}} = 0,$$

for all possible unit vectors $\nu = (\sin \theta \cos \varphi ,\,\,\sin \theta \sin \varphi$, cosθ). This problem can be uniquely solved due to the simplicity assumption for the Riemannian metric and the conditions imposed on *c*(*x*). Upon solving problem (11), (12), we obtain the equation of geodesic lines $\xi = \xi (s,\nu ,y)$, the tangent vector $\eta = \eta (s,\nu ,y)$ to this geodesic line, and the Riemannian distance τ = $\tau (s,\nu ,y)$ along it. Solving the equation $x = \xi (s,\nu ,y)$ with respect to $s,\;\theta ,\;\varphi$, we find $s = s(x,y)$ and $\nu = \nu (x,y)$, that is, the correspondences between a pair of points *x* and *y* and the values of the parameters *s* and $\nu$. The equation of the geodesic line $\Gamma (x,y)$ is given by $\xi = \xi (s,\nu (x,y),y)$, $s \in [0,s(x,y)]$, while the Riemannian distance $\tau (x,y)$, by virtue of relations (11) and (12) for this function, coincides with $s(x,y)$. Note that, according to [10, p. 35] and the above assumption on the smoothness of $c(x)$, it is true that τ^2^ $\in {{C}^{6}}({{\mathbb{R}}^{6}})$.

For $T > 0$, let ${{D}_{T}}$ denote the zone

$${{D}_{T}} = \{ (x,t) \in {{\mathbb{R}}^{4}}|\,{\text{|}}\,t \in [0,T]\} .$$

**Theorem 1.** *Assume that the assumption on the simplicity of the Riemannian metric and conditions* (8) *and* (9) *hold. Then the solution of problem* (4) *is representable in the domain* ${{D}_{T}}$ *as*

13 $$\begin{gathered} u(x,t,y) = \alpha (x,y)\delta (t - \tau (x,y)) \\ \, + [\beta (x,y) + v(x,t,y)]H(t - \tau (x,y)), \quad (x,t) \in {{D}_{T}}, \\ \end{gathered} $$

*where* $H(t)$ *is the Heaviside step function* ($H(t) = 1$ *for* $t \geqslant 0$ *and* $H(t) = 0$ *for* $t < 0$), *the functions* $\alpha (x,y)$ *and* $\beta (x,y)$ *are defined for* $x \ne y$ *by the equalities*

14 $$\alpha (x,y) = \frac{{c(x)\sqrt {{{\rho }_{0}}J(x,y)} }}{{4\pi {{c}^{3}}(y)\tau (x,y)}}\exp \left( { - \frac{1}{2}\int\limits_{\Gamma (x,y)} {{c}^{2}}(\xi )\sigma (\xi )ds} \right),$$

15 $$\beta (x,y) = \frac{{\alpha (x,y)}}{2}\int\limits_{\Gamma (x,y)} {{c}^{2}}(\xi )\left( {\frac{{\Delta \alpha (\xi ,y)}}{{\alpha (\xi ,y)}} - q(\xi )} \right)ds$$

(*where* $\xi \in \Gamma (x,y)$ *is an intermediate integration point and ds is the Riemannian length element*), $v(x,t,y)$ *is continuous for* $t \geqslant \tau (x,y)$, *and* $v(x,t,y) \to 0$ *as* $t \to \tau (x,y)$ *+* 0*.*

We explain below what the function $J(x,y)$ is.

To obtain formulas (14) and (15), we first need to find differential equations and initial conditions for them. These equations can be derived using the common method of calculating the singular and finite amplitudes of the solution of problem (4) (see, for example, [10]). With this aim in view, we must set $v(x,t,y) \equiv 0$ in (13), substitute the resulting equality into (4), and set the coefficients of $\delta '(t - \tau (x,y))$ and $\delta (t - \tau (x,y))$ to zero. As a result, we arrive at the equations

16 $$2\nabla \alpha \cdot \nabla \tau + \alpha (\sigma + \Delta \tau ) = 0,$$

17 $$2\nabla \beta \cdot \nabla \tau + \beta (\sigma + \Delta \tau ) - \Delta \alpha + q\alpha = 0.$$

For a homogeneous medium with parameters $c(x) \equiv c(y)$, $\sigma (x) \equiv 0$, and $q(x) \equiv 0$, it holds that

$$u(x,t,y) = \frac{{\rho _{0}^{{1/2}}\delta (t - {{\tau }_{0}}(x,y))}}{{4\pi c(y){\text{|}}x - y{\text{|}}}},\quad {{\tau }_{0}}(x,y) = \frac{{{\text{|}}x - y{\text{|}}}}{{c(y)}}.$$

Therefore, $\alpha (x,y)$ and $\beta (x,y)$ must satisfy the following conditions as $x \to y$:

18 $$\alpha (x,y) \sim \frac{{\rho _{0}^{{1/2}}}}{{4\pi c(y){\text{|}}x - y{\text{|}}}},\quad x \to y,$$

19 $$\beta (x,y) = O(1),\quad x \to y.$$

Equations (16) and (17) are ordinary differential equations along the geodesic lines $\Gamma (x,y)$. Indeed, due to the first equation in (11) and the accepted notation $\eta = {{\nabla }_{x}}\tau (x,y)$, along the geodesic line $\Gamma (x,y)$ we have

20 $$\nabla \alpha \cdot \nabla \tau = \frac{1}{{{{c}^{2}}(x)}}\frac{{d\alpha }}{{d\tau }},$$

where $d\tau = ds$ is the Riemannian length element. It is convenient to transform the expression for $\Delta \tau (x,y)$ involved in (17) using formula (2.2.35) from [10]. This formula is valid along the geodesic line $\Gamma (x,y)$ and, in our case, has the form

21 $${\text{div}}({{c}^{2}}(x){{\nabla }_{x}}{{\tau }^{2}}(x,y)) = 6 - 2\tau (x,y)\frac{{d\ln J(x,y)}}{{d\tau }},$$

where

$$J(x,y) = \frac{{\partial ({{\zeta }_{1}},{{\zeta }_{2}},{{\zeta }_{3}})}}{{\partial ({{x}_{1}},{{x}_{2}},{{x}_{3}})}}$$

is the Jacobian matrix of the change from the Riemann normal coordinates $({{\zeta }_{1}},{{\zeta }_{2}},{{\zeta }_{3}}) = \zeta$ to the Cartesian coordinates $({{x}_{1}},{{x}_{2}},{{x}_{3}}) = x$. Recall that the Riemann normal coordinates $\zeta$ centered at *y* can be calculated as $\zeta (x,y) = - \frac{{{{c}^{2}}(y)}}{2}{{\nabla }_{y}}{{\tau }^{2}}(x,y)$. From this, we conclude that $J \in {{C}^{4}}({{\mathbb{R}}^{6}})$ and $J(x,x) = 1$.

It follows from (21) that

22 $${{c}^{2}}(x)\Delta \tau (x,y) = 2\frac{d}{{d\tau }}\ln \left( {\frac{{\tau (x,y)}}{{c(x)\sqrt {J(x,y)} }}} \right).$$

In view of (20) and (22), Eq. (16) is transformed into the form

23 $$\frac{d}{{d\tau }}\ln \left( {\frac{{\alpha (x,y)\tau (x,y)}}{{c(x)\sqrt {J(x,y)} }}\exp \left( {\frac{1}{2}\int\limits_{\Gamma (x,y)} {{c}^{2}}(\xi )\sigma (\xi )ds} \right)} \right) = 0,$$

where $\xi = \xi (s) \in \Gamma (x,y)$ and $ds$ is the Riemannian length element. Since $J(x,x) = 1$ and τ(*x*, *y*) ~ ${\text{|}}x - y{\text{|/}}c(y)$ as $x \to y$, equalities (18) and (23) imply (14).

Using (16), we can rewrite Eq. (17) in the form

$$\frac{d}{{d\tau }}\frac{{\alpha (x,y)}}{{\beta (x,y)}} = \frac{{{{c}^{2}}(x)}}{2}\left( {\frac{{\Delta \alpha (x,y)}}{{\alpha (x,y)}} - q(x)} \right).$$

Integration of this equation using (19) yields (15). Obviously, $(\alpha \tau ) \in {{C}^{4}}({{\mathbb{R}}^{6}})$ and $\beta \in {{C}^{2}}({{\mathbb{R}}^{6}})$.

The function $v(x,t,y)$ is a solution of the Cauchy problem

24 $$Lv = f(x,t,y),\quad (x,t) \in {{\mathbb{R}}^{4}};\quad v{{{\text{|}}}_{{t < 0}}} = 0,$$

where the operator *L* is defined by (4) and

25 $$f(x,t,y) = [\Delta \beta (x,y) - q(x)\beta (x,y)]H(t - \tau (x,y)).$$

Equalities (24) and (25) and the finiteness of the speed of sound imply that ${v}(x,t,y) \equiv 0$ for $t < \tau (x,y)$ and, hence, the validity of (13). In addition, the fundamental solution $G(x,t,\xi )$ for (24), that is, the solution of the problem

$$LG(x,t,\xi ) = \delta (x - \xi )\delta (t),\quad (x,t) \in {{\mathbb{R}}^{4}};\quad G{{{\text{|}}}_{{t < 0}}} = 0,$$

has the form

$$\begin{gathered} G(x,t,\xi ) = \rho _{0}^{{ - 1/2}}\alpha (x,\xi )\delta (t - \tau (x,\xi )) \\ \, + {{G}_{0}}(x,t,\xi )H(t - \tau (x,\xi )), \\ \end{gathered} $$

where the function $\alpha (x,y)$ is defined by (14) and ${{G}_{0}}(x,t,\xi )$ is a regular continuous function of its arguments. The solution of problem (24) has the integral representation

26 $$\begin{gathered} v(x,t,y) \\ \, = \int\limits_{{{\mathbb{R}}^{4}}} f(\xi ,t{\kern 1pt} ',y)[\rho _{0}^{{ - 1/2}}\alpha (x,\xi )\delta (t - t{\kern 1pt} '\; - \tau (x,\xi )) \\ \, + {{G}_{0}}(x,t - t{\kern 1pt} ',\xi )H(t - t{\kern 1pt} '\; - \tau (x,\xi ))]{\kern 1pt} {\kern 1pt} d\xi dt{\kern 1pt} '. \\ \end{gathered} $$

It follows from (25) that $f(x,t{\kern 1pt} ',\xi ) = 0$ for $t{\kern 1pt} ' \leqslant \tau (x,\xi )$. Therefore, equality (26) can be transformed into the form

27 $$\begin{gathered} v(x,t,y) = \int\limits_{D(x,y,t)} \text{[}{{\Delta }_{\xi }}\beta (\xi ,y) - q(x)\beta (\xi ,y)]\rho _{0}^{{ - 1/2}}\alpha (x,\xi )d\xi \\ + \int\limits_{\tau (x,y)}^t \int\limits_{D(x,y,t{\kern 1pt} ')} [{{\Delta }_{\xi }}\beta (\xi ,y) - q(x)\beta (\xi ,y)]{{G}_{0}}(x,t - t{\kern 1pt} ',\xi )d\xi dt{\kern 1pt} ', \\ \end{gathered} $$

$$t \geqslant \tau (x,y),$$

where $D(x,y,t)$ is the set

$$\{ \xi \in {{\mathbb{R}}^{3}}\,{\text{|}}\,\tau (x,\xi ) + \tau (\xi ,y) \leqslant t\} ,$$

which is the interior of a Riemann ellipsoid with foci at *x* and *y*. As $t \to \tau (x,y)$, this ellipsoid contracts to the geodesic line $\Gamma (x,y)$ and the volume of $D(x,y,t)$ tends to zero. This implies that ${v}(x,t,y) \to 0$ as *t* → $\tau (x,y)$. The continuity of $v(x,t,y)$ for $t \geqslant \tau (x,y)$ follows from the fact that $\beta (x,y)$ is in the space ${{C}^{2}}({{\mathbb{R}}^{6}})$.

**Remark 2.** When (2) holds, it is valid that

$$\begin{gathered} \alpha (x,y) = \frac{{\sqrt {{{\rho }_{0}}} }}{{4\pi {\text{|}}x - y{\text{|}}}},\quad \Delta \alpha (x,y) = 0, \\ \beta (x,y) = 0,\quad {\text{if}}\quad y \in {{S}_{R}}, \\ 0 < {\text{|}}x - y{\text{|}} \leqslant R - {{r}_{0}}. \\ \end{gathered} $$

We use Theorem 1 to analyze the inverse problem of determining the coefficients in (4) based on data (7). We first note that these data uniquely specify the function $\tau (x,y)$ for all $y \in {{S}_{R}}$ and $x \in {{S}_{R}}(y,{{r}_{0}})$. Indeed, by virtue of (13), for fixed *x* and *y*, we have

28 $$\begin{gathered} \tau (x,y) = \sup \{ \tau > 0\,{\text{|}}\,\Phi (x,t,y) \equiv 0,\,\,t \in (0,\tau )\} \\ \, = {{T}_{0}}(x,y),\quad y \in {{S}_{R}},\quad x \in {{S}_{R}}(y,{{r}_{0}}). \\ \end{gathered} $$

Thereafter, given $\Phi (x,t,y)$, we find $\alpha (x,y)$ and $\beta (x,y)$ for the same *x* and *y*:

29 $$\begin{gathered} \alpha (x,y) = \mathop {\lim }\limits_{t \to \tau (x,y) + 0} \int\limits_0^t \Phi (x,\tau ,y)d\tau , \\ y \in {{S}_{R}},\quad x \in {{S}_{R}}(y,{{r}_{0}}), \\ \end{gathered} $$

30 $$\begin{gathered} \beta (x,y) = \mathop {\lim }\limits_{t \to \tau (x,y) + 0} \Phi (x,t,y), \\ y \in {{S}_{R}},\quad x \in {{S}_{R}}(y,{{r}_{0}}). \\ \end{gathered} $$

These functions are extended to the set ${{S}_{R}} \times {{S}_{R}}$ by using the formulas

$$\begin{gathered} \tau (x,y) = {\text{|}}x - y{\text{|}},\quad \alpha (x,y) = \frac{{\sqrt {{{\rho }_{0}}} }}{{4\pi {\text{|}}x - y{\text{|}}}}, \\ \beta (x,y) = 0\quad {\text{for}}\quad y \in {{S}_{R}},\quad x \in {{S}_{R}}{{\backslash }}{{S}_{R}}(y,{{r}_{0}}). \\ \end{gathered} $$

Then the inverse problem of determining the coefficients $c(x)$, $\sigma (x)$, and *q*(*x*) in ${{B}_{{{{r}_{0}}}}}$ reduces to successively solving the following three problems: (1) finding *c*(*x*) given $\tau (x,y)$, $(x,y) \in {{S}_{R}} \times {{S}_{R}}$; (2) finding $\sigma (x)$ given $\alpha (x,y)$, $(x,y) \in {{S}_{R}} \times {{S}_{R}}$; and (3) finding *q*(*x*) given $\beta (x,y)$, $(x,y) \in {{S}_{R}} \times {{S}_{R}}$.

The first problem is an inverse kinematic one. An estimate for the stability of its solution was derived in [12, 13]. This estimate has the form

$${{\left\| {{{c}_{1}} - {{c}_{2}}} \right\|}_{{{{L}^{2}}({{B}_{R}})}}} \leqslant C{{\left\| {{{\tau }_{1}} - {{\tau }_{2}}} \right\|}_{{{{H}^{2}}({{S}_{R}} \times {{S}_{R}})}}},$$

where ${{c}_{1}}(x)$ and ${{c}_{2}}(x)$ are positive functions generating simple Riemannian metrics, ${{\tau }_{1}}(x,y)$ and ${{\tau }_{2}}(x,y)$ are the corresponding Riemannian distances, and $C = C({{c}_{0}},{{c}_{{00}}})$ is a positive constant depending on the numbers ${{c}_{0}},\;{{c}_{{00}}}$ introduced in (8). The above estimate implies the uniqueness of the solution of the inverse kinematic problem.

The second and third problems lead to an identical problem in integral geometry. Indeed, if the function *c*(*x*) is found, then the geodesic lines $\Gamma (x,y)$ and the function $J(x,y)$ become known. We then uniquely derive from (14) the integrals

31 $$\int\limits_{\Gamma (x,y)} {{c}^{2}}(\xi )\sigma (\xi )ds = g(x,y),\quad (x,y) \in {{S}_{R}} \times {{S}_{R}},$$

where

$$g(x,y) = - 2\ln \frac{{4\pi \alpha (x,y)\tau (x,y)}}{{\sqrt {{{\rho }_{0}}J(x,y)} }}.$$

The problem of determining the function $\hat {\sigma }(x)$ = *c*^2^(*x*)σ(*x*) from (31) is an integral geometry problem. It was studied in [13, 14]. The stability estimate for the solution of this problem inferred in [13, 14] is similar to the estimate for the solution of the inverse kinematic problem with the natural replacement of *c* and $\tau$ by $\hat {\sigma }$ and *g*, respectively. The solution of the integral geometry problem for a family of geodesic lines specified by a simple Riemannian metric is unique. Upon finding $\hat {\sigma }(x)$ and, therefore, $\sigma (x)$, we can calculate the function $\alpha (x,y)$ for any *x* and *y* by applying (14). Based on (15), we then uniquely calculate the integrals

32 $$\int\limits_{\Gamma (x,y)} {{c}^{2}}(\xi )q(\xi )ds = h(x,y),\quad (x,y) \in {{S}_{R}} \times {{S}_{R}},$$

where

$$h(x,y) = \int\limits_{\Gamma (x,y)} {{c}^{2}}(\xi )\frac{{\Delta \alpha (\xi ,y)}}{{\alpha (\xi ,y)}}ds - \frac{{2\beta (x,y)}}{{\alpha (x,y)}}.$$

Consequently, to determine $\hat {q}(x) = {{c}^{2}}(x)q(x)$ from Eq. (32), we have exactly the same integral geometry problem. Upon solving it, we find *q*(*x*) as well. Given this coefficient, we solve problem (6) to find *m*(*x*) and, finally, determine the density $\rho (x) = {{m}^{2}}(x)$.

A result of the previous considerations is the following theorem.

**Theorem 2.** *Under the assumptions of Theorem* 1 *and condition* (2)*, the inverse problem can have only one solution.*

An algorithm for solving the inverse problem is actually described above. In computational respect, it is surely necessary to detail the scheme for constructing solutions of the inverse kinematic problem and the integral geometry problem. Unfortunately, there are currently no sufficiently substantiated efficient algorithms and software programs to solve these problems.
